# PD91 Gaps And Challenges In The Health Technology Assessment Of Genetic And Genomic Applications: A Systematic Review

**DOI:** 10.1017/S0266462324003386

**Published:** 2025-01-07

**Authors:** Leonardo Maria Siena, Antonio Sciurti, Giuseppe Migliara, Claudia Isonne, Giuseppe Di Lorenzo, Maria Roberta De Blasiis, Carolina Marzuillo, Paolo Villari, Valentina Baccolini

## Abstract

**Introduction:**

Evaluating genetic and genomic applications is critical for their adoption in healthcare practice, but this has several challenges, including limited evidence on clinical and non-clinical benefits and rapid development processes. Since these challenges lack comprehensive discussion, this study aimed to identify and analyze all the barriers emerging in the health technology assessment (HTA) of genetic and genomic tests.

**Methods:**

The protocol of this study was registered in the Open Science Framework database. A systematic search of the PubMed, Scopus, and Web of Science bibliographic databases was undertaken to identify all studies that specifically discussed any difficulty, gap, or barrier in the HTA evaluation of genetic or genomic technologies. No restrictions were imposed on HTA domain, study type, or publication date. Gaps and challenges were then grouped into domains outlined in the EUnetHTA HTA Core Model®. A narrative synthesis of the results was performed for each HTA domain.

**Results:**

Of the 457 publications identified, 23 were included. Article designs exhibited considerable diversity, ranging from systematic reviews to semi-structured interviews, and stakeholder involvement varied from universities to national research groups. The challenges we found referred to the general HTA framework or, in most cases, to specific domains. More than 25 percent of the challenges were associated with economic aspects, with cost-effectiveness analysis being the focal point of debate. In addition, clinical effectiveness, in terms of non-health outcomes, lack of evidence, and clinical utility, was also frequently discussed, accounting for 23 percent of the challenges identified.

**Conclusions:**

Our study systematically summarized gaps and barriers in the HTA of genetic and genomic applications, providing a thorough analysis and categorization of these issues. Various challenges surfaced across different domains, notably related to costs, economic evaluation, and clinical effectiveness. There is a need for exhaustive discussions on potential solutions to facilitate and enhance the assessment process for these genetic and genomic applications.

